# Vertical larynx actions and intergestural timing stability in Hausa ejectives and implosives

**DOI:** 10.1515/phon-2023-0052

**Published:** 2024-10-22

**Authors:** Miran Oh, Dani Byrd, Louis Goldstein, Shrikanth S. Narayanan

**Affiliations:** Department of Linguistics, 118557 University of Southern California, Los Angeles, CA, USA; Department of Electrical and Computer Engineering, 118557 University of Southern California, Los Angeles, CA, USA

**Keywords:** non-pulmonic consonant production, timing stability, speech timing, real-time MRI

## Abstract

The current project undertakes a kinematic examination of vertical larynx actions and intergestural timing stability within multi-gesture complex segments such as ejectives and implosives that may possess specific temporal goals critical to their articulatory realization. Using real-time MRI (rtMRI) speech production data from Hausa non-pulmonic and pulmonic consonants, this study illuminates speech timing between oral constriction and vertical larynx actions within segments and the role this intergestural timing plays in realizing phonological contrasts and processes in varying prosodic contexts. Results suggest that vertical larynx actions have greater magnitude in the production of ejectives compared to their pulmonic counterparts, but implosives and pulmonic consonants are differentiated not by vertical larynx magnitude but by the intergestural timing patterns between their oral and vertical larynx gestures. Moreover, intergestural timing stability/variability between oral and non-oral (vertical larynx) actions differ among ejectives, implosives, and pulmonic consonants, with ejectives having the most stable temporal lags, followed by implosives and pulmonic consonants, respectively. Lastly, the findings show how contrastive linguistic ‘molecules’ – here, segment-sized phonological complexes with multiple gestures – interact with phrasal context in speech in such a way that it variably shapes temporal organization between participating gestures as well as respecting stability in relative timing between such gestures comprising a segment.

## 1 Introduction

Ejective and implosive consonants are produced with a glottalic airstream mechanism by initiating airflow in the supralaryngeal vocal tract by means of changes in the vertical larynx position (Catford 1971; Demolin 1995; Greenberg 1970; Ladefoged 1968). These non-pulmonic consonants involve raising or lowering, respectively, of the closed larynx coordinated with an oral constriction formation and release. Although vertical movement of the larynx is one of the characteristics of these non-pulmonic consonants, vertical larynx behavior itself is not unique to this set of speech sounds – e.g., voicing and pitch changes also involve vertical larynx movement, and the vertical aspect of larynx action alone does not fully exhaust the articulatory characterization of the non-pulmonic stops. For example, although phonological contrasts are associated with distinctive patterns of articulatory activity, no clear division is made between implosives and voiced stops in their articulatory characteristics, as they both typically exhibit lowering of the larynx, to decrease oral air pressure and to maintain voicing (Clements and Osu 2002; Kingston 1985; Ladefoged 1968, 1971; Ladefoged and Maddieson 1996).1Voicing can be maintained not only through oral cavity expansion but also through nasal and/or oral leakage during stop closure (Ohala and Solé 2010; Solé 2007, 2014, 2018). It has been hypothesized that there is a continuum between one form of fully voiced stops and true implosives, the latter being produced with a comparatively greater amount of lowering and more rapid descent of the larynx during the coordinated oral gesture than the former (Ladefoged 1971; Ladefoged and Maddieson 1996). However, given that voiced implosives and voiced stops form two distinct linguistic categories as manifested in their phonological distribution (Gallagher 2010; Greenburg 1970; MacEachern 1997; Mackenzie 2009; Newman 2000), the claim that the two classes differ along a continuum without having robust auditory and/or articulatory properties is unsatisfactory.

In addition to the speed and magnitude of larynx raising/lowering, timing of the vertical larynx movement with respect to its coordinated oral constriction formation requires attention. Kingston (1985) raises the question of whether the timing of larynx movement varies to create phonological contrasts. He suggests that larynx movement during ejectives and implosives is “timed so that the larynx is at its highest or lowest point near the oral release, since maneuvers which change the volume of the oral cavity have more profound effects on [oral air pressure] if they are initiated after the oral closure is made” (Kingston 1985:17–18). Moreover, Maddieson and Ladefoged state that the phonemic contrasts between non-pulmonics and their pulmonic counterparts can be manifested by the “differ[ence] in the mode of action of the larynx, or in the timing of laryngeal activity in relation to the oral articulation” (1996: 47). Demolin (1995) shows that oral air pressure may be lowered during the closure of implosives. However, dynamic movement of the vertical larynx gesture for non-pulmonic consonants and its coordination with oral gestures has not been widely studied, and no direct quantified measures of the vertical larynx movement in non-pulmonic stops has been reported.

Widening the consideration of the articulatory characteristics of non-pulmonic consonants, temporal stability between gestures within multi-gesture complexes – contrastive phonological units (such as segments and syllables) that include multiple gestures phased with one another – is relevant. Such complexes must be described in terms of their intergestural timing, as well as composition and parameterization. Ejectives and implosives use a laryngeal airstream mechanism coordinating both oral constriction and vertical larynx movement to create local air pressure changes in the oral cavity. To accomplish this aerodynamic goal, non-pulmonic consonants may require strict timing relations between the oral constriction gesture and the vertical larynx gesture. For instance, in the production of ejectives, the larynx is expected to raise during the time when the oral constriction is sealed so as to yield a large increase in intraoral air pressure. Although there are other factors driving oral air pressure change (e.g., glottalic or laryngeal constriction,2
Dehais-Underdown et al. (2023) and Brandt and Simpson (2021) suggest that ‘glottal’ constriction is just one of many places for airstream initiation in non-pulmonic consonants, and a stricture to build up pressure may occur elsewhere in the larynx, such as ventricular or aryepiglottic constriction. volume expansion), this study focuses on the effect of vertical larynx actions that can potentially contribute to the air pressure change.3Although intra-oral pressure change is an important factor in stops and would be informed by a direct aerodynamic measure of non-pulmonic airstream mechanisms, a modeling study estimating the pressure changes from our MR data is beyond the scope of the current experiment, which focuses on the role of vertical larynx actions and its coordination with the oral gestures.


The current study aims to unveil the role of vertical larynx actions on these non-pulmonic consonants by comparing their movement and timing with the pulmonic counterparts. Each language may show a contribution or the lack thereof of the vertical larynx gesture for ejectives and/or implosives. This study examines whether vertical larynx movement is actually a distinctive and crucial component of non-pulmonic consonants in Hausa.

### 1.1 Instrumental studies on vertical larynx actions

Previous studies measuring vertical movement of the larynx have mainly focused on the relation between larynx height and tone/fundamental frequency (f0). For example, the effects of tonal categories on larynx height were investigated in Gandour and Maddieson’s (1976) cricothyrometer study on Standard Thai and in Wang and Kong’s (2010) X-ray movie data of Mandarin speakers. Vertical positioning of the larynx in singing was examined in Shipp (1975) and Neuschaefer-Rube et al. (1996) using lateral still photographs and Magnetic Resonance Imaging (MRI) data, respectively. Larynx height and its relation to f0 is reported in studies using ultrasound measurements (Hamlet 1980), MRI data (Hirai et al. 1994 **;** Honda et al. 1999), X-ray photographs (Faaborg-Andersen and Sonninen 1960; Lindqvist et al. 1973), a thyrometer (Kakita and Hiki 1976), electroglottography and videofluorography (Laukkanen et al. 1999).

While there is much literature on the relation of vertical larynx position to f0 during speech, only a few research studies have examined laryngeal activities in different segmental articulations.4Although there are many studies on larynx height with respect to vowel height (Hoole and Kroos 1998; Kleiner et al. 2023; Moisik et al. 2019) and rounding (Lindblom and Sundberg 1971; Wood 1986). The following studies investigated larynx mechanisms in sounds other than non-pulmonic consonants. Ewan and Krones (1974) and Riordan (1980) used the thyroumbrometer to measure larynx height in plain stop consonants. Esling and Moisik (2011) used videofluoroscopy with simultaneous laryngoscopy and laryngeal ultrasound to observe larynx height during pharyngeal sounds. Bouarourou et al. (2018) examined geminate and singleton stops in Tarifit Berber using X-ray data. Proctor et al. (2013) investigated laryngeal displacement in a real-time MRI (rtMRI) data during human beatboxing.

As for research specifically involving non-pulmonic consonants such as ejectives or implosives, Shosted et al. (2011) proposed an indirect method for imaging and quantifying the vertical displacement of the larynx using *exterior* electromagnetic articulography (EMA). The estimation of the larynx position of these stops, however, is based on limited data that is not from a native speaker having ejectives and implosives in their language. Bückins et al. (2018) also conducted an EMA study investigating the larynx movement in the production of Georgian ejectives, and they found that ejectives are associated with larger and more upward movement of the skin above the larynx compared to pulmonic sounds. However, both of these studies use an indirect method of estimation from placing EMA sensors on the exterior skin above the larynx (i.e., neck), which has not been validated. Another indirect method of detecting vertical larynx movement in ejectives is introduced in Simpson and Brandt (2019) and Brandt and Simpson (2021), which estimates larynx time functions by the relative amplitudes of the signals from two pairs of electrodes using dual-channel electroglottography (DC-EGG).

The experiment conducted in the present study differs from previous efforts because it directly examines vertical larynx movement coordinated with ejectives and implosives in native speakers’ production using real-time MRI (rt-MRI) of the mid-sagittal vocal tract. Although various methods for quantifying larynx height have been proposed, most of them involve manual handling of the obtained data. For example, in Proctor et al. (2013), laryngeal displacement was measured by manual selection of the end points of a larynx outline. In Honda et al. (1999), vertical larynx position was measured by the rotation angle of the cricoid cartilage, which was traced manually. Moreover, previous studies used either static data or non-dynamic methods that do not permit real-time tracking of the laryngeal movement (Hirai et al. 1994; Honda et al. 1999; Proctor et al. 2013). The current study instead utilizes an automatic tracking method to capture dynamic vocal tract movements, which allows a parallel investigation of oral and non-oral gestures in multi-gesture segments.

### 1.2 Vertical larynx movement in non-pulmonic consonants

Previous studies on ejectives versus pulmonic stops (Brandt and Simpson 2021; Bückins et al. 2018; Simpson and Brandt 2019)have reported differences in the pattern of vertical larynx actions, which needs to be validated by the direct articulatory imaging of the larynx. Traditional accounts of voiceless ejectives, however, are that they involve rapid larynx raising, whereas voiceless pulmonics have not been necessarily associated with vertical larynx movements. In addition, due to the fact that ejective fricatives have leakage at the anterior oral constriction as opposed to the complete oral closure for ejective stops, ejective fricatives may exhibit even larger and/or faster vertical larynx displacement than ejective stops so as to build up the same amount (or sufficient) air pressure and/or to maintain the aerodynamic flow requirements for the generation of turbulence.

Ejectives and implosives, investigated in this study, require specific timing constraints, with narrow temporal intervals. Considering the aerodynamic state of intraoral air pressure change for these non-pulmonic consonants (perceptually to produce bursts with a particular acoustic characteristics), oral and laryngeal gestures would be specifically phased so that the pressure change is maximized for a specific gestural configuration (consider Kingston 1985). Due to this temporal constraint, ejectives and implosives are predicted to have a tighter within-segment timing relation than pulmonic consonants.

This strong temporal cohesion among gestures can be represented by specifying couplings between gestures for segmental gestural molecules. Regardless of the theoretical mechanism for intergestural timing, the temporal control internal to the units must be encoded in the representation of such units to produce systemically cohesive temporal structures in the service of linguistic contrast and morphological structuring of words. Coupled oscillator models can be used to model and predict different intergestural timing patterns in speech production, by representing how gestures are phased with one another in the ‘coupling graphs’ (Goldstein et al. 2007; Nam et al. 2009). A coupling graph is used to represent phonological structures in a gestural planning oscillator model (Goldstein et al. 2007), where each speech unit (e.g., a gesture, a segment, a syllable) is associated with a clock, or planning oscillator, and the clocks can be coupled to one another to create coordination relations. Coupling graphs define whether gestures are coupled together, if so, how and how strongly they are coupled, therefore controlling relative timing between gestures.

**Figure 1:**
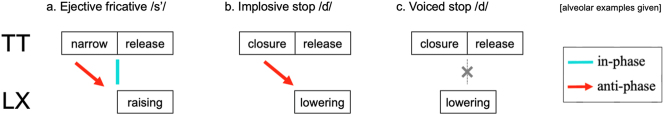
Coupling schema for (a) ejectives, (b) implosives, and (c) voiced plosives.

The gestural schemas presented in Figure 1 demonstrate potential coupling relations between oral and vertical larynx gestures. The coupling relations are hypothesized based on the previous observations of temporal coordination patterns in these consonants. While both ejectives and implosives are expected to exhibit the production of the oral (TT tongue tip shown) constriction gesture before the initiation of the vertical larynx (LX) gesture (indicated by the anti-phase relation in red), this timing relation is not anticipated for pulmonic voiced stops. Although the gestural representation of each category must be validated with experimental data, the coupling structures in Figure 1 illustrate how the same set of gestures could create phonological contrasts via different coupling relations.

The coupling relations assumed in Figure 1 generate predictions on differential stability/variability on timing, in addition to predictions on timing patterns. Studies from Goldstein et al. (2009) and Nam et al. (2009) suggest three means that cause the coupled gestures to be more stable: the coupling can be more stable (i) with greater number of links associated with the gestures, (ii) with a more direct link between the gestures, and/or (iii) with greater coupling strength. Figure 1 illustrates the first of these three means that make the coupling structure more stable. Among the coupling schema presented in Figure 1, Figure 1a depicting ejectives has the most stable timing relations between the oral gesture (TT) and the vertical larynx gesture (LX) because it has greater number of direct links (one in-phase and one anti-phase) among the gestures, followed by the less stable implosives with one direct link (Figure 1b), and by the voiced stops (Figure 1c) with no direct consonant-to-consonant (C-C) coupling relations.5Consonantal gestures for voiced stops (Figure 1c) would instead be indirectly coupled via weak in-phase relations to the tautosyllabic vowel (i.e., consonant-to-vowel [C-V] couplings), although this is not illustrated in Figure 1.


Within the non-pulmonic consonants, ejectives and implosives may also show a difference in their intergestural timing ‘variability.’ The aerodynamic necessity of oral cavity compression and air pressure elevation in ejectives suggests that this timing relation must be highly stable given the necessity for the larynx raising to create a volume compression in the oral cavity (preceding the oral stop’s moment of release). In contrast for implosives, larynx lowering may be associated with (1) lowering pressure just before the release to produce the distinct release characteristics, and (2) expanding oral cavity volume to maintain voicing (as in voiced pulmonic stops). The former is rigidly temporally constrained whereas the latter more flexible coordination is not; this makes it unclear how the temporal coordination with these multiple goals will play out.6Voiceless implosives, although their occurrence is very uncommon across world’s languages, may serve as a direct comparison with voiceless ejectives (Ladefoged 1990). However, no apparent difference in oral air pressure decrease is exhibited between voiced and voiceless implosives (Mc Laughlin 2005). Larynx lowering is thus not locked to a specific timepoint in the course of the oral constriction gesture.

Regarding articulatory distinctions generally between non-pulmonic and pulmonic consonants, it has proven difficult to draw a ‘bright line’ distinguishing the articulation of voiced implosives and voiced stops (though acoustic differences, e.g., in the amplitude envelope of voicing, often exist). This is largely because larynx lowering is not unique to implosives but also for the maintenance of voicing. The current study considers whether the articulatory contrast between these categories might be found in the *temporal* coordination between these pulmonic oral and vertical laryngeal maneuvers. It is hypothesized that the coordination of gestures in non-pulmonic consonants is critical, whereas the coordination between supralaryngeal and laryngeal gestures for pulmonic consonants (e.g., an oral constriction gesture and a downward larynx gesture for voicing) is likely to be more loosely coupled in time. (Though Löfqvist and Yoshioka [1981, 1984] do report stable timing in the face of perturbation for vocal fold adduction and oral closure of pulmonic stops.).

By investigating temporal relations between gestures of non-pulmonic and of pulmonic consonants, the current study aims to reveal coordination and coupling structures among gestures that comprise a multi-gestural segmental unit. The investigation of timing stability will provide useful information for the representation of coupling structures for these phonological complexes with multiple gestures.

### 1.3 Predictions

Hausa, mainly spoken in Nigeria and the Republic of Niger, is examined because both ejectives and implosives, as well as their pulmonic counterparts, occur in this language (Jaggar 2001; Schuh and Yalwa 1993). Hausa ejectives are produced in three places of articulation – alveolar ejective fricatives (/(t)s’/), velar ejective stops (/k’/), and labio-velar ejective stops (/k^w^’/) – which can be compared with pulmonic consonants, /s/, /k/, and /k^w^/, respectively. Hausa has bilabial and alveolar voiced implosives (/ɓ/ & /ɗ/), as well as their plain counterparts: voiced bilabial and alveolar stops /b/ and /d/.

Three areas of investigation are addressed regarding articulatory dynamics of non-pulmonic consonants in Hausa. The study examines the velocity profile of the vertical laryngeal gesture (Hypothesis A), intergestural timing of the vertical larynx-oral coordination (Hypothesis B), and stability in intergestural timing under prosodic variations between non-pulmonic and pulmonic stops (Hypothesis C).

The first hypothesis serves as reaching a quantitative confirmation that the rtMRI images examined with the data analysis protocol described below can provide an informative account of the articulatory patterns of laryngeal movement for the speakers of Hausa and how they differentiate non-pulmonic consonants from pulmonic consonants in the language. We hypothesize that ejectives and implosives show raising and lowering, respectively, of the larynx to a greater degree than found in a corresponding, paired pulmonic consonant (Clements and Osu 2002; Kingston 1985; Ladefoged 1968, 1971; Ladefoged and Maddieson 1996). Specifically, Hypothesis A is tested as below:

**Hypothesis A:**

Non-pulmonic consonants show larger vertical larynx movement than their pulmonic counterparts.

Turning next to intergestural timing in non-pulmonic consonants, the coordination of the laryngeal raising or lowering with the oral constriction must occur while the oral closure is in place if a significant oral pressure differential is to result. That is, both ejectives and implosives require oral constriction formation to occur before the initiation of the vertical larynx action. On the other hand, for pulmonic consonants, no such temporal requirement is necessary between the oral gesture and any accompanying vertical larynx movement. Pulmonic voiced stops may involve larynx lowering movement so as to facilitate voicing during closure via volume expansion,7A low larynx position helps in maintaining voicing by increasing transglottal pressure (Westbury and Keating 1986). but the vertical larynx movement need not occur at the moment of oral closure. Therefore, the sequential timing between oral and vertical larynx movements is predicted to occur in the production of ejectives and implosives but not precisely in the production of pulmonic consonants. Thus, the following hypothesis is entertained:

**Hypothesis B:**

Oral gestures precede vertical larynx gestures in ejectives and implosives, while oral and vertical larynx movements are not temporally constrained in pulmonic counterparts.

As for the stability and variability in temporal coordination, the proposed coupling schema in Figure 1, together with the articulatory descriptions, leads to Hypothesis C.

**Hypothesis C:**

The temporal lag between oral closure and vertical larynx onset gestures in ejectives is more stable than the lags in implosives, and pulmonic consonants have the most variable intergestural timing.

The variability in timing between gestural actions is examined by analyzing intergestural timing across prosodic variations. We especially focus on whether the measures of variability in articulatory actions and timing can differentiate the three linguistic categories, separating ejectives from implosives, and non-pulmonic consonants from pulmonic ones (excluding voiceless consonants as they do not have a target specified for vertical larynx movement). Empirical findings from these vertical larynx-oral actions have further import for the phonological representations of these multi-gesture segments, as well as for models of internal coupling graphs.

With the ability to quantify the MRI data automatically, it is possible not only to investigate the relative timing of the gestures composing these consonants, but also to compare how tightly or loosely coupled to each other they are. Investigating such coordination structures and their timing variability in multi-gesture speech segments illuminates whether ‘stability/variability’ in relative timing is linguistically relevant, with potential linguistic consequences of coupling structures for sound change, learnability, or perceptual recoverability.

## 2 Methods

### 2.1 Subjects

The subjects are three native Hausa speakers (S1-S3; ranging from 24 to 30 years old) residing in the United States at the time of the experiment. They are all from Northern Nigeria. The subjects were instructed to read aloud the target sentences written in Hausa orthography, which were presented on a projection screen one at a time. The subjects spoke lying supine on a MR scanner bed and were able to read the prompts from inside the scanner using a mirror, without moving their heads. The total recording time including calibration was about one hour.

### 2.2 Data acquisition

**Figure 2:**
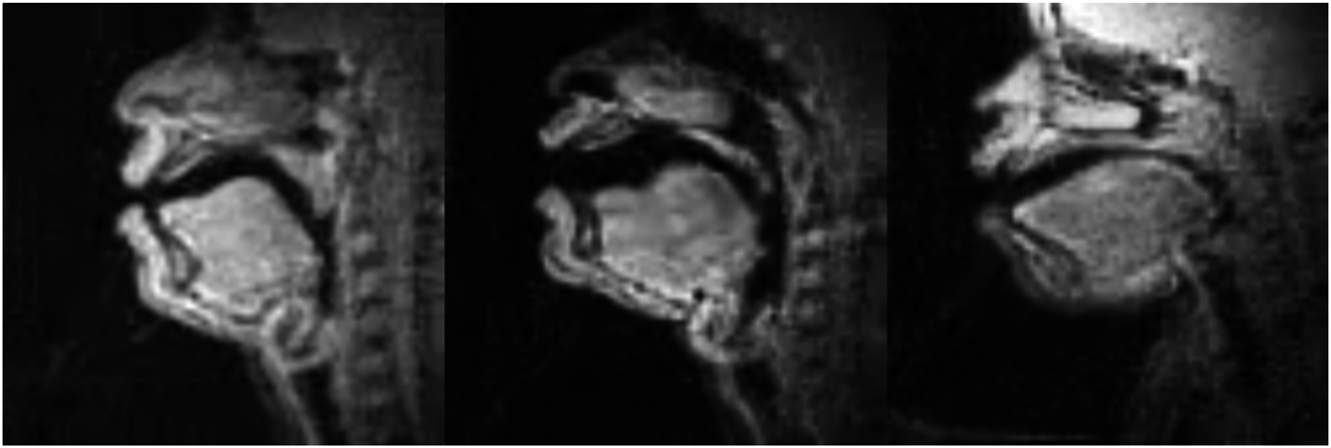
Vocal tract mid-sagittal images of three subjects (S1, S2, and S3, respectively).

MRI data of the mid-sagittal vocal tract and audio data were simultaneously acquired using a real-time MRI protocol developed for research on speech production (Narayanan et al. 2004). Data were acquired at Los Angeles County Hospital on a 1.5 T scanner with gradient amplitude of 4.0 G/cm and 10.5 G/cm/ms slew rate. A 13-interleaf spiral gradient echo pulse sequence was used. Each spiral is acquired over 6.004 ms (repetition time (TR)); therefore, every image comprises information spanning 13 × 6.004 = 78.052 ms. Image data were acquired at a rate of 12 frames/second, an imaging field of view (FOV) of 200 × 200 mm, and a flip angle of 15 degrees. Slice thickness was 6 mm, located mid-sagittally; image size was 84 × 84 pixels yielding a spatial resolution in the sagittal plane of 2.4 mm. The videos were reconstructed with a 2-TR sliding window giving an effective frame rate of 83.3 frames/s (=1/(2 × TR) = 1/(2 × 6.004 ms)), enabled by constrained reconstruction (Lingala et al. 2016, 2017). Sample MRI images of the midsagittal plane for three subjects are presented in Figure 2.

Audio was simultaneously recorded inside the scanner at a sampling frequency of 20,000 Hz while the subject was imaged. The audio signals were synchronized with the MR video signal for data analysis per published protocols (Lingala et al. 2016, 2017; Narayanan et al. 2004). The recorded speech was enhanced by the acoustic denoising method developed in Vaz et al. (2018) and was synchronized with the reconstructed dynamic MR images. The subjects wore foam ear plugs to attenuate scanner noise but were still able to communicate with the experimenters outside the scanner room via an in-scanner intercom system.8A portion of the current MRI data are presented in a poster introducing larynx tracking methodologies (Oh and Lee 2018; Oh et al. 2017), and partial analyses are reported in Oh et al. (2018) and in the first author’s unpublished PhD dissertation (Oh 2021). The current study is expanded from former studies by providing the full analysis of the data, with special attention to the articulatory representation of ejectives and implosives.


### 2.3 Materials

Target stimuli obtained for data analysis were voiceless velar and labialized velar ejective stops (/k’, k^w^’/), alveolar ejective fricatives (/s’/), and voiced bilabial and alveolar implosive stops (/ɓ, ɗ/) in Hausa. In addition, pulmonic voiceless stops and fricatives (/k, k^w^, s/) and voiced stops (/b, d/) were collected; stimuli are given in Table 1. These ten target consonants were located at two prosodic conditions: phrase-initial and phrase-internal positions. An example of two prosodic conditions is given in (1). For phrase-initial conditions, target consonants (e.g., /k/ in [1a]) are located at the beginning of the intonational phrase (IP), where IP boundaries are created by a pause induced by commas. For phrase-internal conditions (1b), words with target consonants (an object noun) immediately follow a phrase-initial word.

**Table 1:** Target consonants in Hausa.

	Bilabial	Alveolar	Velar	Labio-velar
Plosive	b	d	k	k^w^
Implosive	ɓ	ɗ		
Ejective		s’	k’	k^w^’
Fricative		s		
Nasal	m	n

(1)

a.

**Table:** 

Phrase-initial
*Fàɗa: sàu ɗaya,* ***kà:za:*** *shine kalmà: à Hausa*.^9^
‘Say once, chicken is the word in Hausa.’

9 Note. Low tone is marked by a grave accent (`), and long vowels are marked by a colon (:). In the presentation to subjects, however, tone and duration are not marked, as is standard in written Hausa.

b.

**Table:** 

Phrase-internal
*À yànzu, biyà:* ***kà:za:*** *kàmar̃ kalmà: à Hausa*.
‘Right now, read aloud chicken as a word in Hausa.’

Each carrier sentence is 14 syllables long, and the target word is located six syllables from the beginning of the sentence. Each target consonant is at the onset of a bi-syllabic word having a Low-High tone sequence. All target consonants were preceded and followed by the vowel /ɑ/. Each target item was repeated 7 times in a randomly ordered list. A total of 168 tokens (12 consonants × 2 prosodic conditions × 7 repetitions) were collected for each speaker. (Nasals are not directly compared with other consonants in this study but are used to illustrate general larynx actions).

### 2.4 Data analysis

#### 2.4.1 Region-of-interest analysis for oral gestures

**Figure 3:**
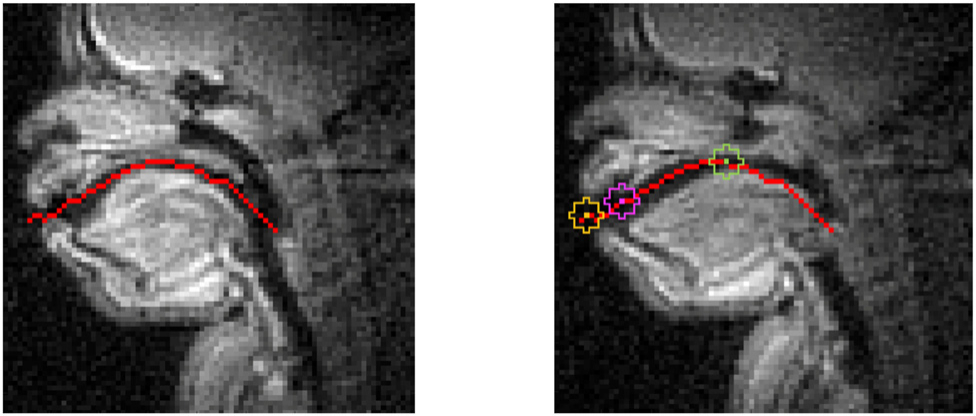
Vocal tract mid-line from automatic calculation (left) & ROI overlayed image (images taken from S3; right: image of a speaker producing a vowel /ɑ/, with regions of interest [LABIAL in yellow, CORONAL in pink, and DORSAL in green from left to right]).

Two techniques were used to obtain information on articulatory timing of the supralaryngeal and laryngeal (vertical) gestures from the real-time MRI video recordings. First, a Region-of-Interest (ROI) technique (Bresch et al. 2010; Lammert et al. 2013a; Proctor et al. 2011) was used to track supralaryngeal constriction formation over time – specifically lips, tongue tip, and tongue body movements involved in the production of bilabial, alveolar, and velar consonants, respectively. To do this, a mid-line of the vocal tract was calculated by selecting pixels with the highest standard deviation over time (Figure 3: left). The regions placed along this mid-line most effectively capture the fluctuation of pixel intensities (Blaylock et al. 2016; Lammert et al. 2013b). Three pseudo-circular regions with a radius of three pixels were manually placed along the automatically derived mid-line over the locations of oral constriction formations (Figure 3: right). The first region (LABIAL or ‘LAB’) was selected around the front of the lower lip so that the region covers the movement of the lower lip when it is most protruded and so that the boundary of the region touches the upper lip. The second region (CORONAL or ‘COR’) was placed over the location of tongue tip constriction, that is, with its top edge at the alveolar ridge and immediately posterior to but non-overlapping with the labial region. The final region (DORSAL or ‘DOR’) was placed at the front-end of the soft palate (velum) to capture movement for tongue rear (velar) constriction gestures.

**Figure 4:**
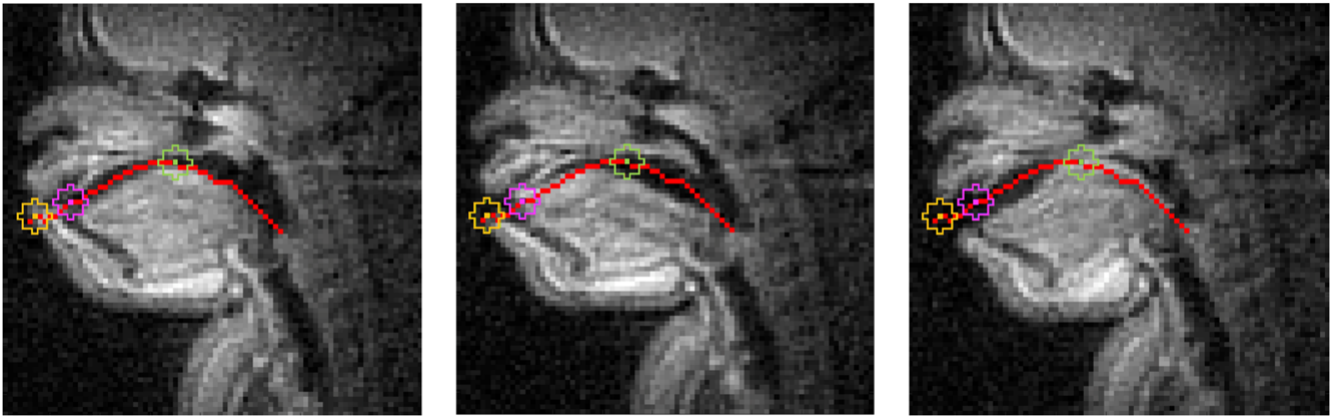
ROI-overlayed images of a speaker producing /b/ (LAB), /d/ (COR), and /k/ (DOR) from left to right (images taken from S3).

Examples of the constrictions produced by labial, tongue tip, and tongue body gestures are presented in Figure 4. The average pixel intensity in each region (a semicircular region with 261 sq mm) was calculated frame-by-frame. The pixel intensity values over time provide time series reflecting articulator motions, where higher mean pixel intensity indicates a greater amount of tissue in the region (Lammert et al. 2013b), as the active articulator of interest forms a constriction at the passive articulator along the upper surface of the vocal tract. Each of these circular ROIs are full when a corresponding oral constriction is mostly formed (Figure 4). These time series were smoothed using a locally weighted linear regression technique with the kernel width of *h* = 0.9 (Lammert et al. 2013b; Proctor et al. 2011).

#### 2.4.2 Centroid tracking analysis for non-oral gestures

The second technique used for the articulatory analysis – specifically for laryngeal movement – is the centroid tracking technique developed for this project (Oh et al. 2017, 2018, 2019; Oh and Lee 2018; see also Tilsen et al. 2016).10
Oh and Lee (2018) demonstrates the performance of centroid tracking analysis and its usefulness in tracking non-constriction movement gestures. Code and scripts are available at https://github.com/miranoh/ACT
 In contrast to the oral constriction formation gestures, action of the velum and larynx (involved in the production of oral/nasal articulation and ejectives/implosives, respectively) may not be analyzable as the formation of a constriction in a specific location.

**Figure 5:**
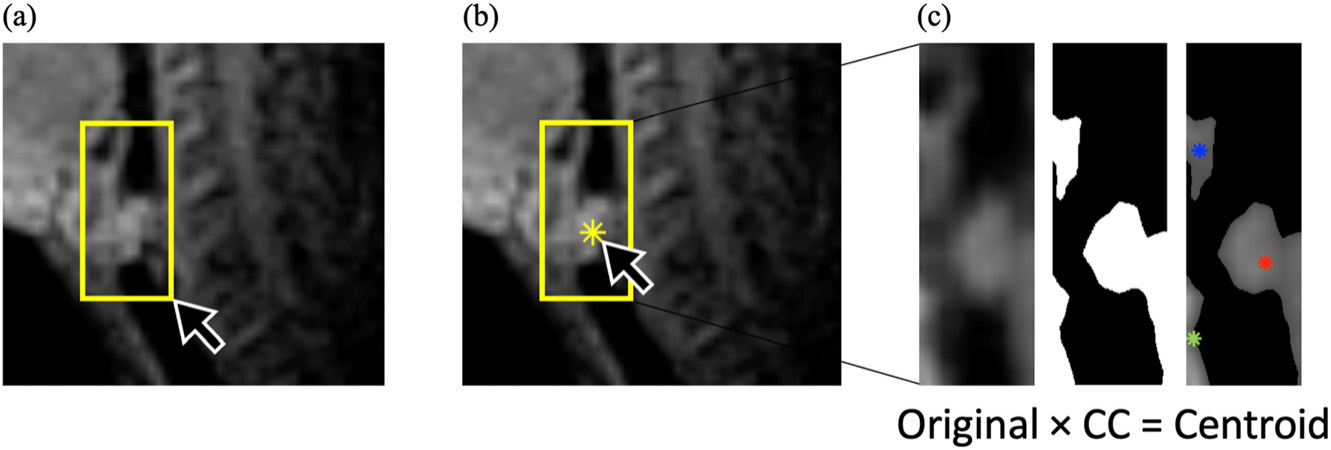
Pre-processing steps for automatic centroid tracking: (a) manually select the rectangular VTR for larynx, (b) select seed to capture only the larynx object, (c) get intensity-weighted centroids based on pixel intensity of three connected components (CC) inside the VTR. The closet centroid from the seed is chosen as the first centroid for larynx (Oh and Lee 2018; images adapted from Oh and Lee 2018).

Vertical movement of the larynx was measured by tracking the time-varying pixel intensity centroid (i.e., intensity-weighted average spatial position) of manually selected rectangular Vocal Tract Regions (VTR). For the larynx, a fixed VTR (‘LX’) was selected for each subject based on the location of cervical vertebra – defined from the bottom line of the 2nd cervical vertebra to the bottom line of the 4th cervical vertebra and having the posterior side of the larynx region placed at the rear pharyngeal wall. The dimension of the larynx VTR was 10–20 mm in width and 40 mm in height, which was ‘tall’ enough to include the highest and the lowest position of the larynx inside the region for each speaker.11Larynx (‘LX’) VTR sizes for each speaker: Speaker A (14.5 × 40 mm), Speaker B (17.5 × 40 mm), and Speaker C (11.5 × 40 mm). Once the VTR was defined, an initial seed is selected anywhere on the object of interest (see Figure 5 and Oh and Lee 2018). The intensity-weighted centroid of each connected component in the VTR was calculated, and the centroid that was closest to the seed was selected as the centroid of the first frame. In the following frames, the centroid closest to the previous centroid was set as the current centroid. This process was undertaken to capture only the movement of an object that is of interest and to prevent other objects that come into the VTR from impinging on the calculation of the larynx centroid. Aside from the seed selection, the subsequent centroids are tracked automatically.

**Figure 6:**
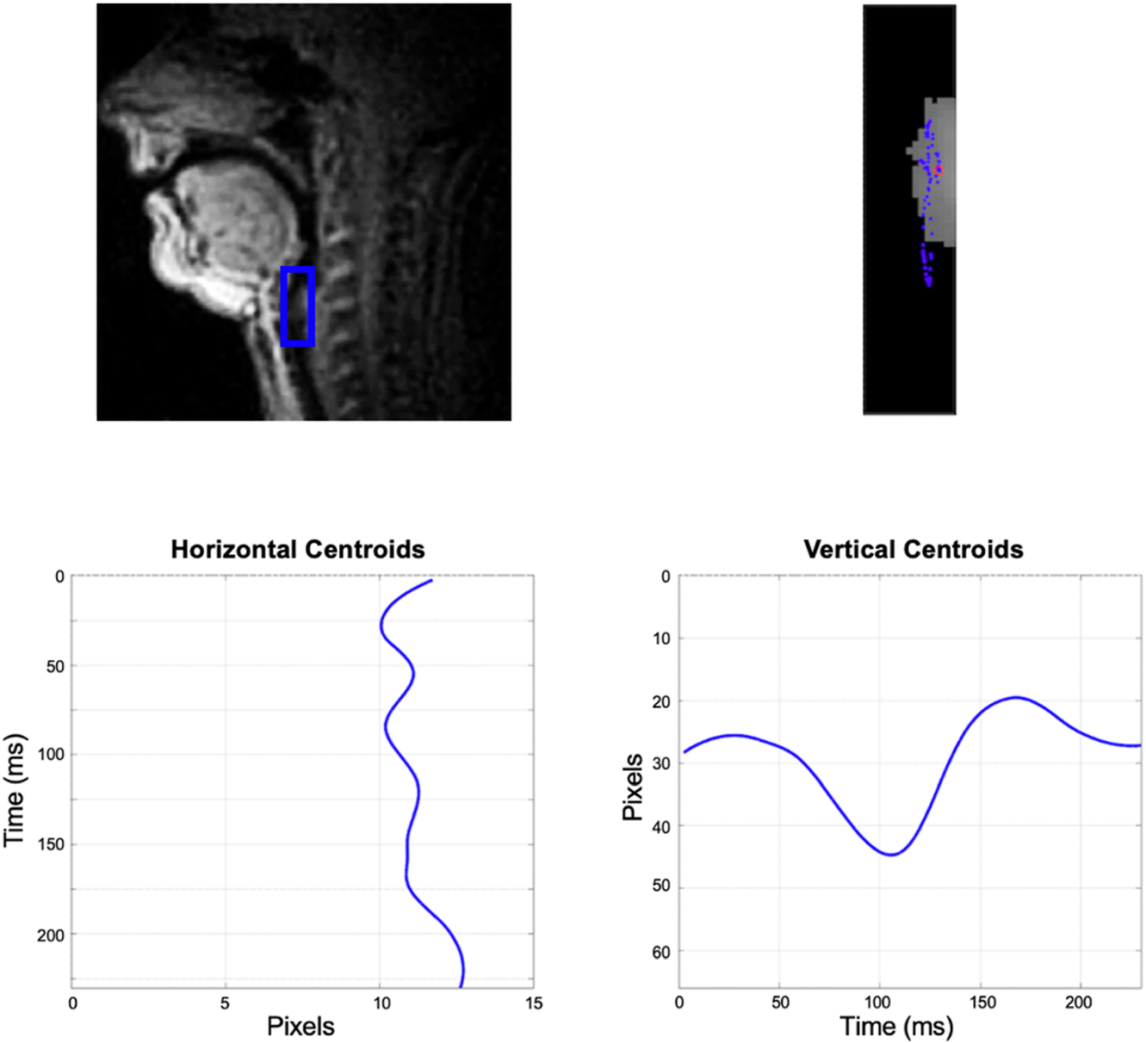
The centroid tracking output of the production of a VCV sequence /ɑɠɑ/.

By way of exemplifying the algorithm, Figure 6 shows the output for a trained phonetician producing the VCV sequence /ɑɠɑ/ with an intervocalic voiced velar implosive (from the USC-IPA dataset; Toutios et al. 2016). The VTR selected for the larynx is shown as a blue box on the top-left plot and on the top-right plot. The vertical centroid plot (bottom-right) of the larynx region exhibits a clear lowering of the larynx (starting at about 50 ms and ending around 150 ms, as we expect to see in the production of implosives). The centroid tracking technique can also capture velum raising/lowering movement as well as the horizontal larynx movement (the bottom-left plot in Figure 6) that may be associated with vertical larynx actions. This tool enables obtaining kinematic profiles of various articulatory movements that have not previously been satisfactorily quantifiable. In the current study, we will focus on the vertical movement of the larynx.

The vertical position of the centroid for the larynx gesture is retrieved as the resulting signal from the MRI data. To reduce noise and intensity fluctuations, all signals were smoothed using a locally weighted quadratic polynomial regression model (i.e., a loess smoothing) with a local span of 30 datapoints.

#### 2.4.3 Measurements

**Figure 7:**
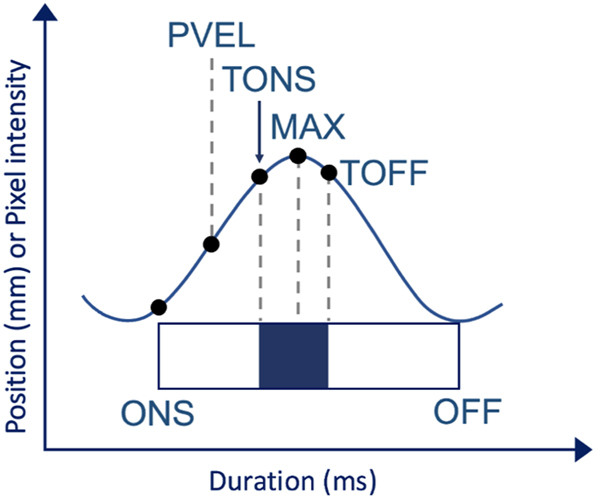
Temporal landmarks of a schematic gesture (based on pixel intensity for oral [labial, coronal, & dorsal] gestures [cf. 2.4.1] & centroid position for LX gestures [cf. 2.4.2]). The landmarks are defined by velocity thresholds of the gestural movement.

Based on the trajectories obtained from the ROI technique and the centroid tracking technique, the temporal landmarks of the articulatory actions used in producing the target Hausa consonants were calculated using the find_gest algorithm (Tiede 2010) (Figure 7).

Movement onset (ONS) was defined to be the point at which velocity reached 20 % of its first maximum velocity for the movement towards the target. Peak velocity (PVEL) was defined at the maximum velocity point during the movement. Target attainment (TONS) was identified as the timepoint after maximum velocity and before maximum extremum position at which velocity fell below a 20 % threshold of the first maximum velocity. The maximum displacement of the gesture (MAX) was defined at the velocity minimum closest to the movement’s peak intensity/centroid weighting. The target offset (TOFF) was calculated at the point following maximum displacement at which velocity increased above the 20 % threshold of the gesture’s maximum velocity for the movement away from the target. Derived variables of intragestural durations and intergestural lag were quantified using the temporal landmarks in Figure 7. The dependent variables in this study are:

– LX displacement (in mm; change in position between MAX & ONS)
– LX extremum (in mm; absolute position of the larynx at its spatial maximum)
– Temporal lags (in ms; LX_ONS minus Oral_TONS or Oral_ONS) (Positive lag values indicate that the larynx movement onset follows the oral landmark.)
  - Interval from oral closure target to vertical larynx movement onset
  - Interval from oral movement onset to vertical larynx movement onset
– LX duration (in ms; time from vertical larynx movement onset [LX_ONS] to movement offset [LX_TOFF])

Tokens were necessarily omitted for individual dependent variables when the gestures in the target word were not captured with the find_gest algorithm.12Number of items (out of 14 tokens for each consonant) without quantifiable vertical larynx movement, and thus omitted from analysis, are represented inside the parentheses. S1: /m/ (8), /n/ (7), /ɗ/ (1), /b/ (3), /d/ (2), /k/ (3), /k^w^/ (1), /s/ (1), /k^w^’/ (2); S2: /m/ (2), /n/ (2), /b/ (3), /k/ (1), /k^w^/ (2), /k^w^’/ (1); S3: none. For example, utterance-initial LAB gestures for bilabial stops were not likely to be identified when a speaker had closed lips during the pause between utterances.

For statistical testing on timing variability across prosodic variations, linear mixed effects regression models are used with subjects, items, and prosodic boundaries as random effects with Tukey’s post-hoc pairwise comparison tests for LX magnitude and intergestural timing patterns,13For all linear mixed effects regression models and Tukey’s post-hoc comparisons, the Kenward-Roger’s method, a more conservative degrees of freedom method than the default Satterthwaite’s method, was used for *lmerTest* package in R (Kuznetsova et al. 2017). and Levene’s tests for homogeneity of variance (HOV) with means were used. To test for significant differences in variance between groups of different consonant types, a modified version of Levene’s tests for homogeneity of variance is used, which tests the equality of the population variances by carrying out an analysis of variance of the absolute values of deviations of observations from the group median (rather than using the group mean, as proposed in the original Levene’s test [Levene 1960]14Performing analysis of variance across deviations from the median instead of the mean is reportedly more robust to departures from normality (Anderson 2006). For example, the Brown-Forsythe test is an extended version of the Levene’s test in that the analysis of variance is carried out on the absolute deviations about the median (Brown and Forsythe 1974). The modified Levene’s tests used in the current analysis are equivalent to the Brown-Forsythe tests for equality of variances.). The *LeveneTest* function in the package *car* (companion to applied regression; Fox 2016; Fox and Weisberg 2019) implemented in R is used to determine whether there is equality of variance on intergestural timing between ejectives and implosives, and between voiced implosives and voiced stops. Testing differences in variance is supplemented with tests of coefficient of variation, which is particularly useful when comparing groups with different means, measures, or values. Specifically, modified signed likelihood ratio tests (M-SLRT) for the equality of coefficients is selected, which is considered superior to SLRT in terms of controlling the Type I error rates (Diciccio et al. 2001) and performs better than asymptotic tests when the sample size is relatively small (Krishnamoorthy and Lee 2014; Marwick and Krishnamoorthy 2019). The level of statistical significance was set as *p* < 0.05.

## 3 Results

### 3.1 Vertical laryngeal activity

Ejectives and implosives are predicted to show larger and faster vertical movement of the larynx than their pulmonic counterparts (Hypothesis A). To test this hypothesis, the actions of larynx raising in ejectives and larynx lowering in implosives are examined individually. The two prosodic conditions (phrase-initial and phrase-internal) are pooled for statistical analyses so as to investigate the overall characteristics of vertical laryngeal actions on non-pulmonic and pulmonic consonants. In Table 2, overall descriptive statistics are presented for LX displacement and extrema per speaker. In addition to the contrast between voiceless and voiced consonants, with voiceless ones having an upward LX movement and voiced ones downward LX movement, vertical larynx displacement is slightly larger in ejectives compared to their pulmonic counterparts.

**Table 2:** Descriptive statistics for LX displacement (mm) and LX extremum (mm) (mean (sd)).

		S1	S2	S3	Total
Voiceless ejectives	Count	40	41	42	123
	LX displacement	5.33 (4.5)	6.81 (4.2)	3.33 (2.1)	5.14 (4.0)
	LX extremum	25.87 (4.6)	25.9 (4.4)	30.54 (2.9)	27.46 (4.6)
Voiceless consonants	Count	37	38	42	117
	LX displacement	2.89 (3.7)	6.65 (3.3)	2.29 (1.8)	3.89 (3.6)
	LX extremum	25.52 (5.8)	23.43 (2.3)	29.61 (2.9)	26.3 (4.6)
Voiced implosives	Count	27	28	28	83
	LX displacement	−1.92 (3.5)	−4.79 (4.4)	−1.64 (1.5)	−2.79 (3.6)
	LX extremum	21.84 (3.8)	18.12 (4.9)	26.94 (3.2)	22.3 (5.5)
Voiced consonants	Count	23	25	28	76
	LX displacement	−0.62 (4.5)	−3.62 (5.6)	−1.91 (2.8)	−2.08 (4.5)
	LX extremum	20.68 (5.2)	18.24 (6.1)	26.27 (4.1)	21.92 (6.4)

#### 3.1.1 Larynx raising

The magnitude of larynx raising is reflected in two dependent variables: larynx displacement and extremum. The displacement measure indicates the change in vertical position from the movement onset to extremum, whereas the absolute vertical extremum position indicates where within the pre-defined vocal tract region the larynx is located at its highest/lowest for a gesture. The production of voiceless consonants does not have target for larynx raising movements and are thus considered here as the control to examine whether voiceless ejectives comparably have larger larynx raising magnitude.

**Figure 8:**
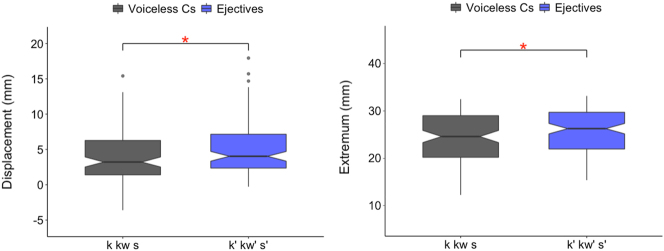
Larynx raising displacement (left) and larynx extremum (right). (The lower and upper box hinges correspond to the first and the third quartiles [i.e., the inter-quartile range, or IQR], the whiskers extend to the smallest and the largest value within the 1.5 × IQR, and the notches extend 1.58 × IQR/sqrt[n].).

In Figure 8, vertical larynx actions involved in voiceless consonants and voiceless ejectives are compared in terms of vertical displacement and absolute extremum position. The linear mixed effects model shows that ejectives have larger displacements and higher extremum positions of the larynx than pulmonic consonants (displacement: F(1,209.61) = 8.145, *p* < 0.01*; extremum: F(1,191.5) = 8.341, *p* < 0.01*).

**Figure 9:**
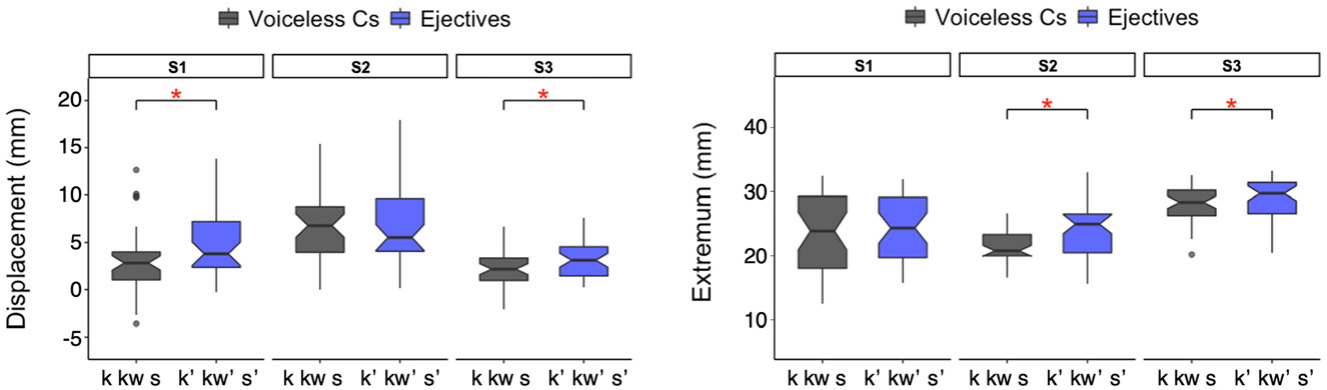
Larynx raising displacement (left) & extremum (right) for individual speakers.

Individual speaker results for larynx magnitude (Figure 9) show that the significant differences between voiceless consonants and ejectives are found in larynx displacement for S1 and S3 and in larynx extremum for S2 and S3.15Larynx displacement for S1: F(1,69.002) = 7.401, *p* = 0.008*; for S2: F(1,65.959) = 0.096, *p* = 0.758; for S3: F(1,70.972) = 7.850, *p* = 0.007*; Larynx extremum for S1: F(1,61.173) = 0.307, *p* = 0.582; for S2: F(1,71.827) = 9.485, *p* = 0.003*; for S3: F(1,57.944) = 6.487, *p* = 0.014* This shows that the speakers differentiate either the larynx displacement (S1) or the larynx extremum (S2), or both (S3) to produce contrast between plain and ejective consonants, which all results in the common general pattern of ‘bigger’ vertical larynx movement for ejectives.

**Figure 10:**
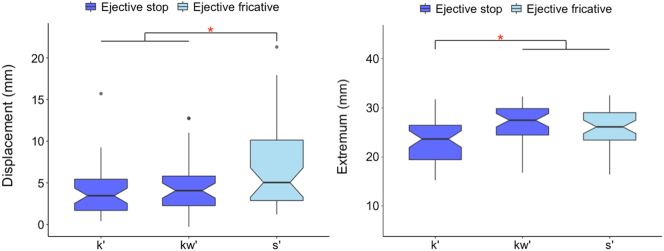
Larynx raising displacement (left) & extremum (right) in ejective stops and fricatives.

Let’s zoom in to ejectives and compare different manner of articulation – ejective stops (/k’, k^w^’/) versus ejective fricatives (/s’/). There is a main effect of segment on larynx raising displacement (F(2,96.141) = 7.474, *p* < 0.001*; Figure 10: left), with Tukey’s post-hoc tests suggesting that the larynx raises more in the production of ejective fricatives compared to that of ejective stops (/s’/ vs. /k’/: *t*(96.4) = 3.765, *p* < 0.001*; /s’/ versus /k^w^’/: *t*(108.1) = 2.624, *p* = 0.027*; /k^w^’/ versus /k’/: *t*(86.6) = 1.06, *p* = 0.541). The effect of segment on larynx extrema is also significant (F(2,87.152) = 10.325, *p* < 0.001*; Figure 10: right), but the distinction is not due to the manner class contrast; rather velar ejective stops have lower extremum positions than labio-velar ejective stops (/k’/ vs /k^w^’/: t(76.9) = 4.316, *p* < 0.001*) and alveolar ejective fricatives (/k’/ vs. /s’/: t(88.1) = 3.252, *p* < 0.01*), but no distinction in larynx extrema is found between the latter two segments with different manner of articulation (/k^w^’/ vs. /s’/: *t*(100.6) = −1.043, *p* = 0.551).

#### 3.1.2. Larynx lowering

**Figure 11:**
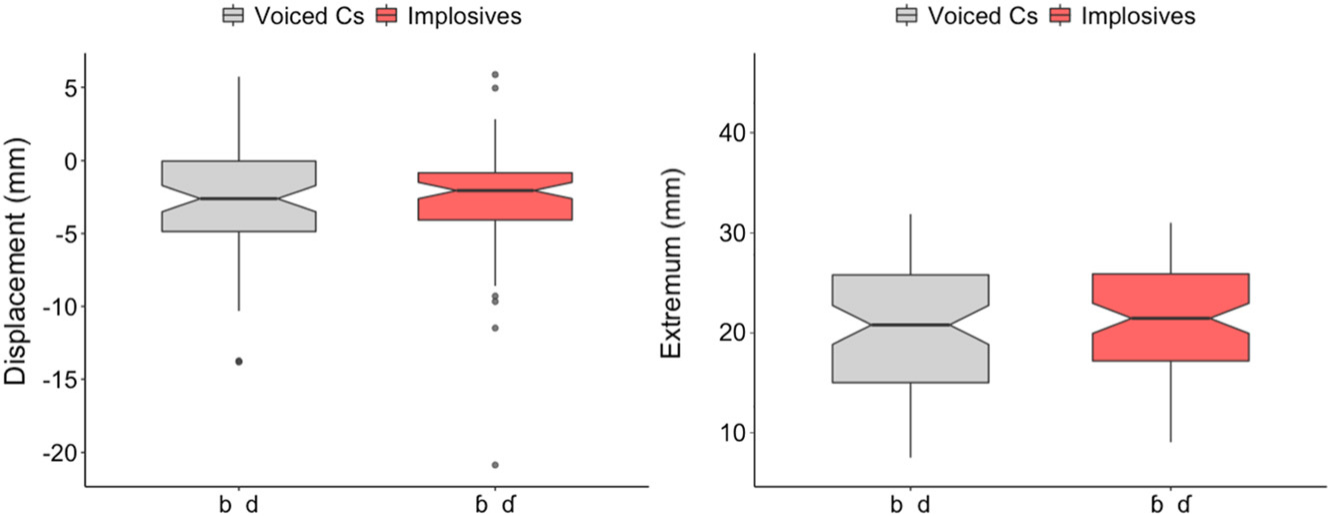
Larynx lowering displacement (left) & larynx extremum (right). (See Figure 8 for other details).

Larynx lowering magnitude is measured by lowering displacement and by extremum (i.e., the absolute position in the VTR when the larynx is maximally lowered). Voiced pulmonic consonants (/b, d/) and voiced implosives (/ɓ, ɗ/) are compared; it was predicted that larynx lowering magnitude will be greater in implosives than in voiced pulmonic consonants (Hypothesis A). Findings, however, show that neither larynx lowering displacement (F(1,134.13) = 2.211, *p* = 0.139) nor extrema (F(1,119.9) = 0.265, *p* = 0.608) differentiates these two classes (Figure 11).

**Figure 12:**
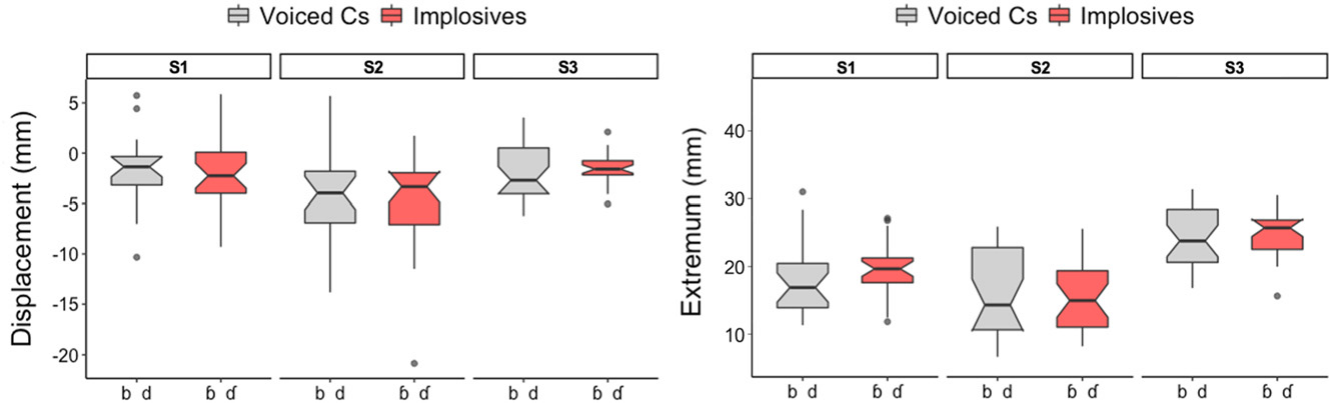
Larynx lowering displacement (left) & extremum (right) for individual speakers.

This lack of distinction in the degree of lowering between plain voiced stops and voiced implosives is consistent in the results of individual speakers (Figure 12). No speaker (S1-S3) exhibits differing degrees of larynx lowering (either in displacement or in extremum) between their production of voiced pulmonic consonants and implosives. This implies that the relatively well-accepted previous description of this phonological contrast stating that implosives have larger downward larynx movement than pulmonic consonants (Clements and Osu 2002; Ladefoged 1971; Ladefoged and Maddieson 1996) is not supported in our spatial data on larynx lowering.

**Figure 13:**
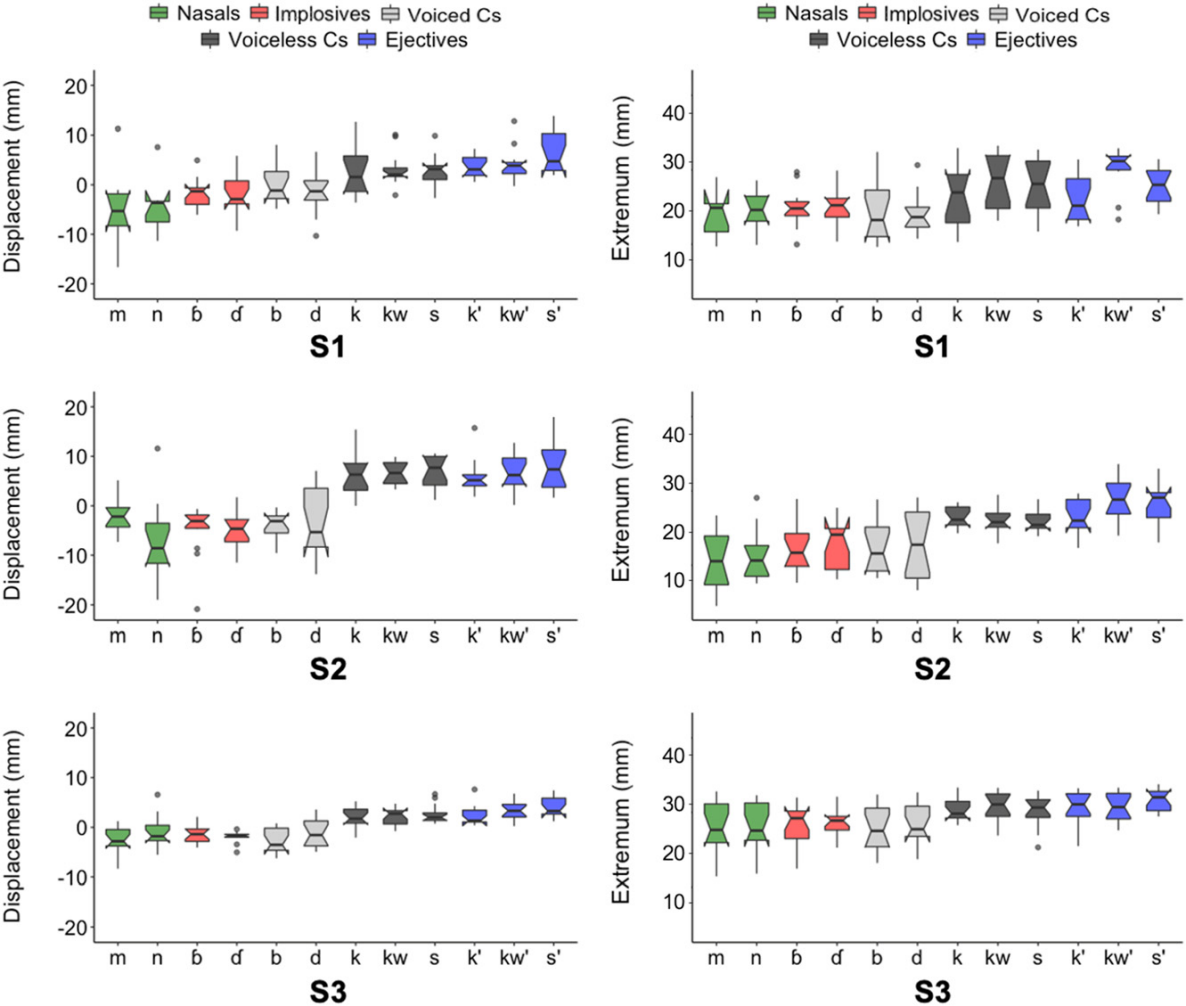
Larynx displacement (left) & larynx extremum (right) for individual speakers.

Figure 13 presents overall larynx displacement and extremum data for the individual speakers, including nasal consonants (in green) as well as pulmonic and non-pulmonic stops and fricatives. In this figure, we observe a variable trend of larynx displacement increasing in the order of nasals < voiced implosives < voiced stops < voiceless consonants < voiceless ejectives. However, we observe individual differences: for Speaker 2, the laryngeal pattern is more or less categorical between voiced and voiceless consonants, and for Speaker 3, there appears to be no difference between nasals, implosives and voiced stops.

### 3.2 Larynx-oral coordination

In this section, the *coordination* of the vertical larynx gesture and the oral gesture is compared between non-pulmonic consonants (ejectives and implosives) and pulmonic consonants (Hypothesis B), followed by the investigation of *stability/variability* in coordination (Hypothesis C). Mean temporal lags (onset lags [LX_Onset minus oral_Onset] and oral target to LX onset lags [LX_Onset minus oral_Target; LX_ons_ – Oral_tar_]) for each speaker are presented in Table 3. Voiceless consonants are not included here, as no active specification is assumed for intergestural timing in such segments. Overall, we observe smaller onset lags in pulmonic consonants, and close-to-zero oral target to LX onset lags in non-pulmonic consonants.

**Table 3:** Descriptive statistics for vertical larynx-oral temporal lags (ms) (mean (sd)).

		S1	S2	S3	Total
Voiceless ejectives	Count	40	41	42	123
	Onset lag	70.55 (90.2)	43.63 (81.5)	78.63 (92.5)	64.33 (88.7)
	LX_ons_ – Oral_tar_	−43.53 (67.3)	−66.48 (71.4)	−31.44 (82.7)	−47.05 (75.0)
Voiced implosives	Count	27	28	28	83
	Onset lag	22.69 (95.7)	102.50 (53.2)	77.62 (115.0)	68.14 (96.4)
	LX_ons_ – Oral_tar_	−75.16 (101.6)	0.85 (48.6)	−15.01 (121.3)	−29.23 (99.8)
Voiced pulmonics	Count	23	25	28	76
	Onset lag	−20.87 (160.3)	−11.53 (119.0)	−35.16 (136.3)	−23.06 (137.3)
	LX_ons_ – Oral_tar_	−132.62 (136.8)	−122.0 (114.3)	−145.82 (140.0)	−133.99 (129.7)

#### 3.2.1 Non-pulmonic versus pulmonic consonants: temporal organization

**Figure 14:**
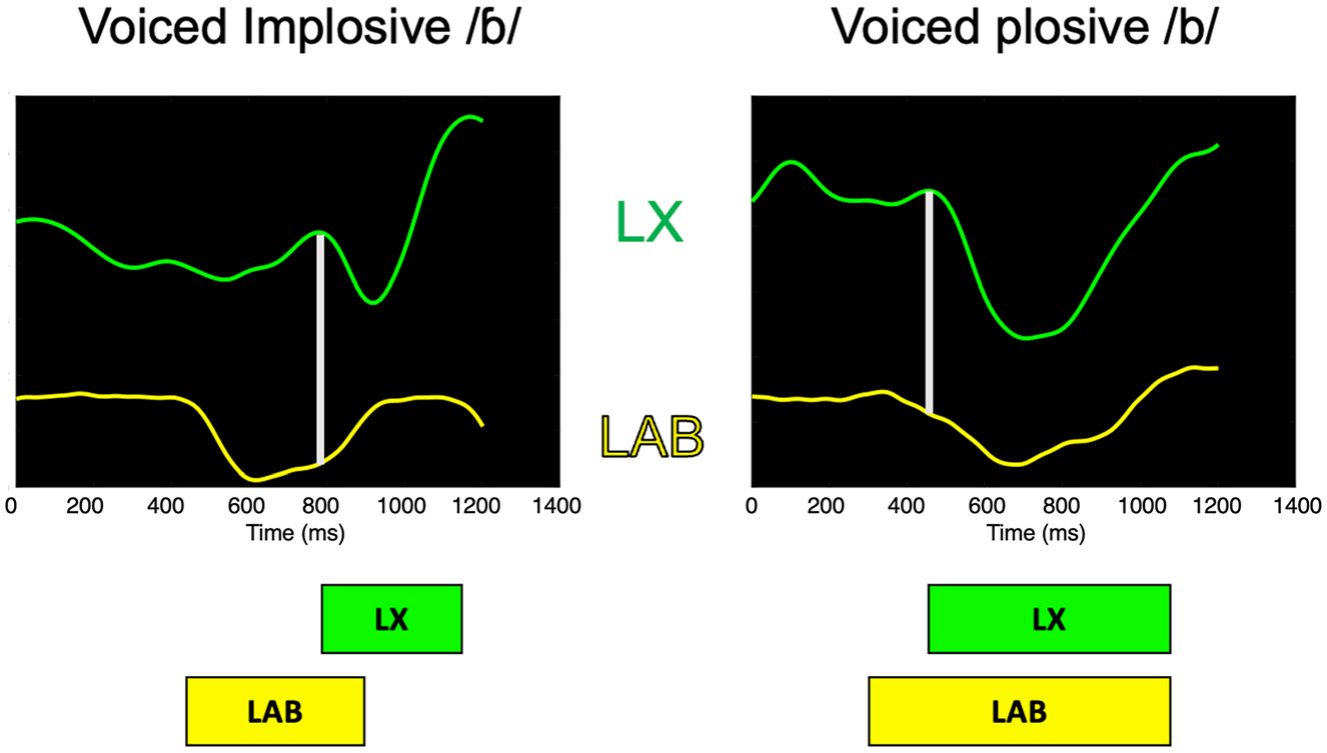
Sample trajectories for voiced implosives (left) and voiced plosives (right). The boxes in the bottom represent the movement onset to the offset of the LX and the oral LAB gestures. Vertical lines are drawn as reference lines marking the onset of the LX gesture (cf. Figure 7 for temporal landmarks).

For Hypothesis B we test whether oral gestures precede vertical larynx gestures in ejectives and implosives, while oral and vertical larynx movements exhibit a freer coordination in pulmonic counterparts. Figure 14 presents sample gestural trajectories for voiced implosives and voiced plosives. This shows that the LX gesture begins to lower after the oral constriction (LAB) has been achieved for implosives, whereas the LX gesture and the coordinated oral gesture begin roughly simultaneously for plosives.16In Figure 14, the trajectories for the oral LAB gestures go down with more constriction and go up as the lip closure releases.


**Figure 15:**
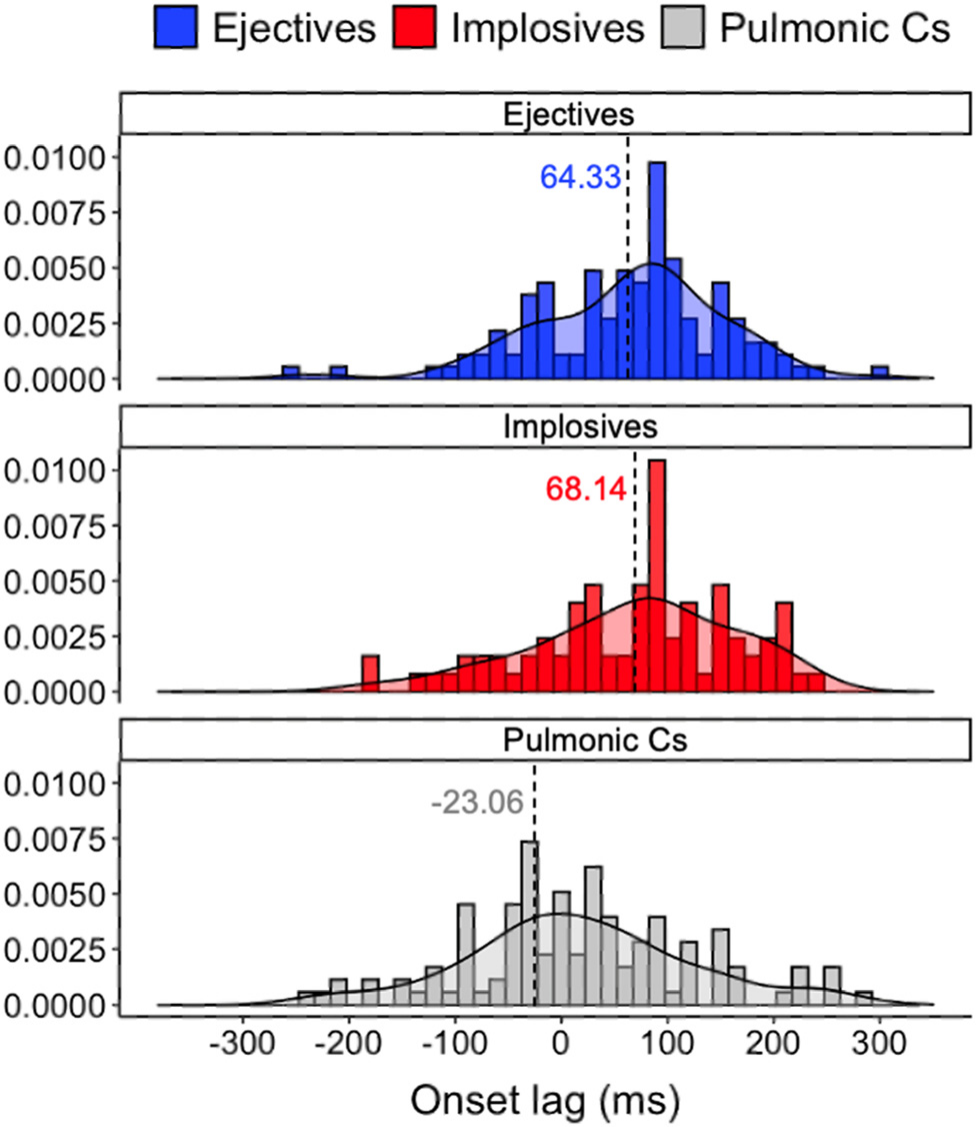
Histogram and distribution of onset lags for ejectives, implosives, and pulmonic consonants (dashed lines indicate median).

Onset-to-onset lags (from oral movement onset to vertical larynx movement onset) are a useful index of how the two gestures are phased to each other. Results Figure 15 shows that for pulmonic consonants, onset lags are near-zero (median: −23.06 ms), while ejectives and implosives have slightly positive onset lags (median: for ejectives 64.33 ms, for implosives 68.14 ms).17In the current dataset, oral closure duration for (non)pulmonic stops ranges from 200 to 400 ms and LX raising/lowering duration ranges from 200 ms to 600 ms (cf. Oh 2021). A positive lag indicates that the vertical larynx action gesture begins after the initiation of the oral closure gesture. There is a main effect of consonant type on onset lags (F(2,361.25) = 20.119, *p* < 0.001*), and pairwise comparison tests reveal that non-pulmonic consonants have longer onset lags than pulmonic consonants (Tukey’s: ejectives vs. pulmonic Cs: t(360) = 5.436, *p* < 0.001*; implosives versus pulmonic Cs: t(353) = 4.887, *p* < 0.001*; ejectives versus implosives: t(377) = −0.093, *p* = 0.995).

The observed temporal lags (i.e., oral onset to LX onset lags) indicate that pulmonic and non-pulmonic consonants have fundamentally different timing patterns: non-pulmonic consonants are produced with larynx raising/lowering starting only after the initiation of the oral closure gesture, while oral constriction formation and vertical larynx action are synchronous in the production of pulmonic consonants. In addition to distinctive temporal coordination patterns, the variability in temporal coordination between the oral and the vertical larynx gesture is explored in the next section.

#### 3.2.2 Non-pulmonic versus pulmonic consonants: *timing variability*

In this section, stability across prosodically-induced variation in non-pulmonic consonants for two intergestural lags are examined: first, the onset-to-onset lag (i.e., onset lags), which is the interval from oral constriction movement onset to larynx raising/lowering onset, and second, the onset-to-target lag measuring the interval from larynx raising/lowering onset to the target achievement of the oral closure. It is predicted that temporal lags are more variable in implosives than in ejectives, and that the lags are more variable in voiced plosives than in voiced implosives (Hypothesis C). These predicted patterns of timing variability are observed in the token-to-token variability. We further investigate how flexible or rigid the intergestural timing is in the face of prosodic perturbation at varying phrase boundaries. To measure relative stability/variability in timing, covariance analyses are conducted between intergestural timing and gestural duration.

##### 3.2.2.1 Token-to-token variability

Hypothesis C states that the temporal lag between oral closure gesture and the coordinated vertical larynx gesture in ejectives and implosives is predicted to be stable, but for pulmonic airstream mechanism consonants with no specific aerodynamic constraints in the timing between oral closure and vertical larynx movement, intergestural timing is expected to be more variable. Phrase boundary conditions are pooled (phrase-initial and -internal) to allow for a wider sample of timing flexibility across prosodic contexts. This is possible due to the previous observation of this dataset exhibiting prosodic boundary effects on oral duration and on LX magnitude, showing that gestures in ejectives and implosives undergo lengthening or strengthening under prosodic variations (Oh 2021).

**Figure 16:**
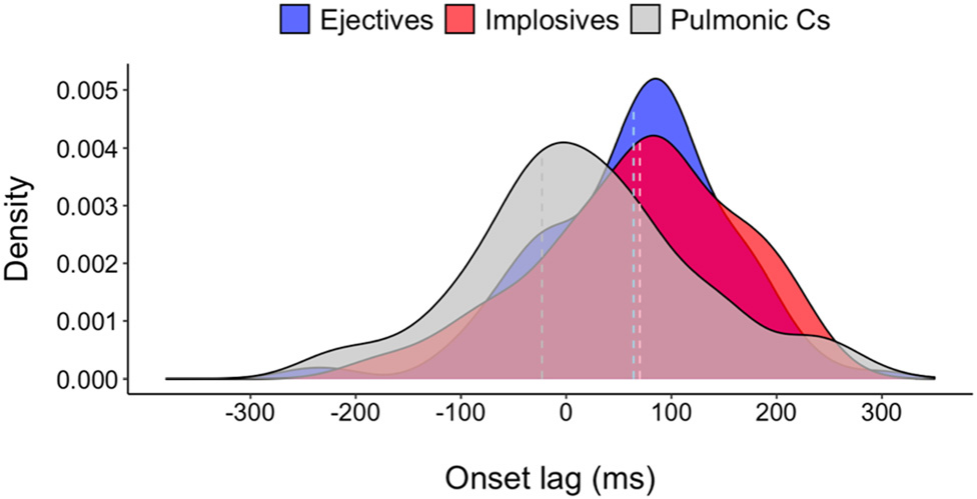
Density distribution for onset lags in pulmonic consonants, ejectives, and implosives (dashed lines indicate median).

Figure 16 shows density plots for different consonant types overlayed to compare the kernel density estimate, or a smoothed histrogram, in intergestural timing distribution. In addition to differential temporal patterns between pulmonic consonants and non-pulmonic consonants, there is a significant difference in timing variability, pulmonic consonants showing more variable onset lags than the lags in ejectives and implosives (F(1,380) = 8.355, p < 0.01*). Timing variability in onset lags is also significantly different within the non-pulmonic consonants, ejectives exhibiting less timing variability than implosives (ejectives vs. implosives: F(1,103) = 7.968, *p* < 0.01*).

**Figure 17:**
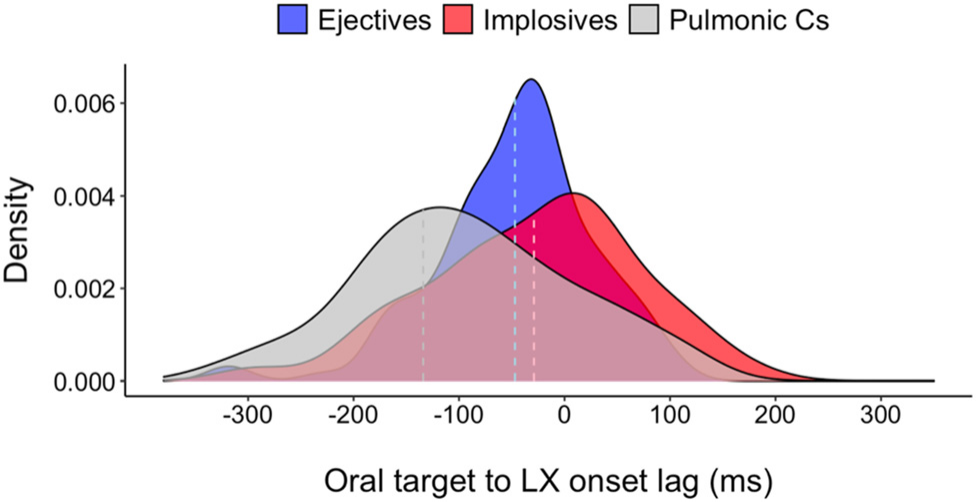
Density distribution for oral target to vertical larynx onset lags in pulmonic consonants, ejectives, and implosives (dashed lines indicate median).

This differential stability in timing between non-pulmonic and pulmonic consonants is also seen for the lags between LX onset and oral *target* achievement. Figure 17 plots the distribution of oral target to LX onset lags. In addition to a difference in temporal patterns between non-pulmonic and pulmonic consonants (non-pulmonics have a near-zero lag, whereas pulmonics have a negative lag18This is statistically confirmed in linear mixed effects models showing a main effect of consonant type on oral target to larynx onset lag (F(2,364.48) = 25.622, *p* < 0.001*). Tukey’s post-hoc pairwise comparisons indicate that non-pulmonic consonants have smaller negative lags than pulmonic consonants, while no lag difference is found between the pairs of non-pulmonic consonants (ejectives vs. pulmonic Cs: t(363) = 5.52, *p* < 0.001*; implosives versus pulmonic Cs: t(356) = 6.142, *p* < 0.001*; ejectives versus implosives: t(381) = -1.182, *p* = 0.465).), Levene’s tests show that pulmonic consonants show a more variable timing than non-pulmonic consonants for this temporal lag (F(1,380) = 10.816, *p* < 0.01*), and this is clearly seen in the qualitative data visualized in Figure 17. For the two non-pulmonic consonants, ejectives and implosives, overall timing variability across prosodic conditions indicates that ejectives show less variability in onset-to-target lags than implosives (Levene’s test for ejectives vs. implosives: F(1,204) = 8.631, *p* < 0.01*).

The observations from the two intergestural lag measures – onset lags and oral target-to-LX onset lags – provide clear support for Hypothesis C, that ejectives have more stable temporal lags than implosives.

##### 3.2.2.2 Correlations between timing and duration

In this section, correlations between individual gestural duration and intergestural timing are examined to test whether intergestural timing is relatively stable in the presence of variations in individual gestural duration. Again, it is expected that implosives show stable intergestural timing across variations in individual gestural duration, whereas voiced consonants’ timing is affected by changes in gestural duration.

**Figure 18:**
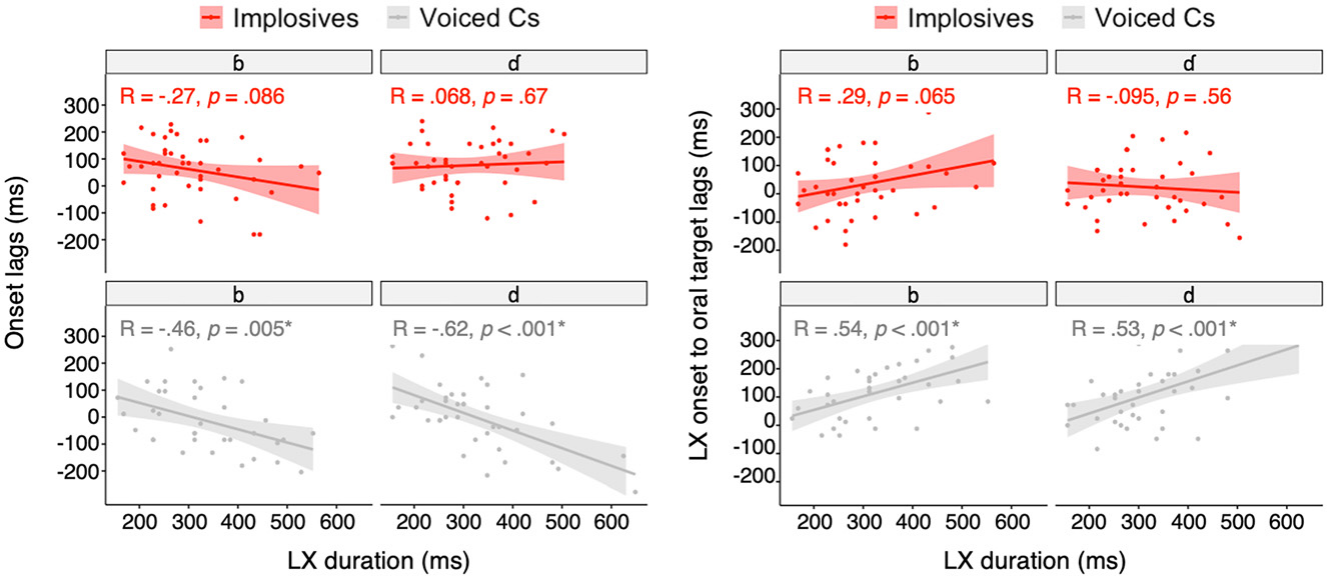
Correlation graphs for intergestural timing and LX duration (left: onset lags & LX duration; right: LX onset to oral target lags & LX duration).

The correlation results for onset lags show that there is a significant negative correlation between the onset lag and LX duration for voiced stops (/b/: *R = *−0.46; /d/: *R* = −0.62), while onset lags remain stable across variations in vertical larynx duration for voiced implosives (Figure 18: left). Similarly, LX onset to oral target lags and LX duration show significant positive correlations for voiced stops (/b/: R = 0.54; /d/: R = 0.53), whereas no correlation (positive or negative) is found for onset-to-target timing versus LX duration for implosives (Figure 18: right). In other words, for voiced stops, as the duration of the larynx action lengthens, larynx lowering begins earlier relative to the oral closure onset (negative onset lags as a function of LX duration), and oral closure target achievement is delayed relative to the larynx lowering onset (positive onset-to-target lags as a function of LX duration). However, neither intergestural lag changes much in voiced implosive stops regardless of the duration of the vertical larynx movement.

These different patterns of covariance relations provide evidence that implosives and voiced pulmonic stops have different coordination constraints such that oral closure target is closely synchronized with the onset of the larynx lowering for implosives, whereas oral target may be coordinated to another specific timepoint within larynx movement interval (e.g., larynx lowering target) for voiced stops, and thus these temporal lags are more affected by changes in LX duration. Additional statistical results indicate how the temporal lags are represented distinctively in implosives, but not in voiced stops: Shapiro-Wilk normality tests for univariate distribution show that onset-to-target lags (W = 0.938, *p* > 0.345) as well as onset lags (W = 0.972, *p* > 0.068) follow univariate normality for implosives, but each of these individual variables for voiced stops does not exhibit normal distribution (onset-to-target lags: W = 0.934, *p* < 0.01; onset lags: W = 0.967, *p* < 0.05), rejecting the null hypothesis and supporting a multivariate distribution. Furthermore, coefficients from the Linear Discriminant Analysis (LDA) show that the onset-to-target lag (LD1 coefficient: 0.0141) has a greater effect size than the onset lag (LD1 coefficient: 0.0059) when discriminating between implosives and voiced stops (using R package *lda*). In sum, findings on these two intergestural timing variables (onset lags and LX onset to oral target lags) suggest that implosives’ vertical larynx onset is timed with the coordinated oral gesture, and this timing relation is independent of the duration of vertical larynx actions.

## 4 Discussion

This study illuminates the dynamics of the vertical larynx gesture in Hausa ejectives and implosives and the temporal coordination with larynx action with associated oral constriction gestures, which have not previously been directly studied in articulation. Findings suggest that only ejectives, but not implosives, have larger vertical larynx movement than their pulmonic counterparts (Hypothesis A partially supported), temporal arrangements are different in non-pulmonic versus pulmonic consonants (Hypothesis B), and the oral-to-LX timing is the least variable in ejectives, followed by less variable implosives and the most variable voiced stops, respectively (Hypothesis C).

The results on vertical larynx action demonstrate that the LX magnitude is larger in voiceless ejectives (which involve larynx raising) than in voiceless pulmonic consonants, which have no apparent basis for larynx raising.19Except possibly to contribute to supralaryngeal cavity shrinkage for devoicing (Westbury 1983). Individual speaker results indicate that speakers may use different mechanisms to increase raising magnitude, either by starting from a lower beginning position so that the larynx can move further up or by ending higher, or a mix of both. Both serve to decrease the volume of oral cavity and presumably create the raised air pressure that is critical for the class of ejective consonants. In addition, ejective fricatives exhibit larger larynx raising displacement (but not higher extremum) than ejective stops. This mechanism of a larger upward movement appears to serve the need for creating a sufficiently large increase in oral air pressure for the ejective fricatives, which, with their lack of a complete seal in the oral cavity and their consequent venting to create turbulent flow, require extensive larynx raising to build up enough oral pressure needed for both the laryngeal airstream mechanism and for the turbulent airflow. It is, however, possible that the result may come from the ejective fricatives having a more anterior position than ejective stops (alveolar /s’/ vs. velar /k’, k^w^’/), therefore requiring more vertical larynx movement (or other lingual movements) to achieve the same amount of intraoral pressure change.

In contrast, between voiced implosives and voiced pulmonic stops, no significant difference is found in larynx lowering. In contrast to previous claims describing contrasts in lowering magnitude between implosives and plain voiced consonants (Ladefoged 1971; Ladefoged and Maddieson 1996), such a spatial magnitude difference is not exhibited in the current data. Our findings are in fact consistent however with previous observations that implosives in Hausa and other languages such as Owerri Igbo do not necessarily exhibit a drop in intraoral pressure (Clements and Osu 2002). Ladefoged (1976) describes that voiced bilabial implosives in Owerri Igbo, compared to voiced bilabial stops, do not have increase nor decrease in oral pressure during the closure, the air pressure being approximately the same inside and outside the oral seal (i.e., the mouth). Instead, implosives may be more adequately characterized by a lack of increase in oral air pressure, though this must be validated with aerodynamic data. Such pressure differences, if they exist, might possibly result from the different coordination pattern of oral constriction with larynx lowering, as discussed below.

Turning next to temporal lags between vertical larynx and oral gestures in the Hausa stops, the results show that in the non-pulmonic consonants the larynx raising/lowering action begins after the oral closure movement onset and slightly before the achievement of the oral closure target. Recall that in implosives and pulmonic voiced stops the degree of larynx lowering is similar, suggesting that the phonological contrast between the two stop classes may have other articulatory foundations, such as the relative timing between oral constriction and vertical larynx movement or the details of the glottal adduction and/or laryngealization.20
Lindsey et al. (1992) points out that Hausa implosives may in fact be categorized as ‘laryngealized stops’ in that they show an aryepiglottal constriction that voiced plosives lack. Nevertheless, we observe clear, coordinated larynx lowering for Hausa implosives, regardless of the potential presence of an aryepiglottal gesture, which has a distinctive coordination with the oral stop release. Among many possibilities, the current findings suggest that a distinction between the two stop categories in Hausa is apparent in their segment internal temporal lags. Both the vertical larynx onset to oral target lags and the larynx-oral onset lags show that for implosives the larynx lowering starts after the onset of oral constriction formation, beginning around the time of oral closure achievement. In contrast, the vertical larynx action and the oral closure gesture begin roughly simultaneously for voiced pulmonic stops. The later lowering of the larynx in implosives is expected in that the acoustics of implosives are typically characterized by increasing voicing amplitude as the release approaches (Demolin 1995; Jessen 2002; Kulikov 2010; Ladefoged and Johnson 2014; Ladefoged and Maddieson 1996), as compared to the typical amplitude die-out of voicing during the closure of pulmonic voiced stops. In the production of implosives, as the larynx lowers after the closure, the volume of the vocal tract increases, keeping supralaryngeal pressure from increasing, which allows voicing of the implosives to be maintained with the same or increasing amplitude throughout the closure (Lindau 1984; Russell 1997). For the Hausa voiced stops, on the other hand, the larynx lowers before the closure is formed, and the vocal tract size gradually decreases during the closure. In turn, the supralaryngeal pressure increases and voicing eventually dies out. One possible result of these different coordination patterns would therefore be expected to be different acoustic/auditory characteristics at stop release.

While it seems to be the case that vertical larynx movement or the intergestural timing is one of the controlled speech motor actions necessary in the representation and production of ejectives and implosives, the vertical laryngeal gesture as a variable has not usually been implemented in articulatory models or has been only represented statically in terms of larynx height (see e.g., Browman and Goldstein 1989; Goldstein 1980; Maeda 1990; Ménard et al. 2007; Mermelstein 1973). Additionally glottal adduction or laryngealization, and potential pitch changes must also necessarily be represented in articulatory control models; indeed for the consonants examined here the former is requisite to successfully achieve the aerodynamic requirements of these consonants. Still, as gestural sequencing is shown here to be a crucial factor in differentiating one category from another (implosives and voiced stops in this case), a better understanding of the dynamics of the vertical laryngeal activities and associated gestures’ timing relations in non-pulmonic consonants is necessary in developing articulatory models and representations, as well as refining traditional phonetic accounts for how ejectives and implosives are initiated via complex laryngeal action. The current findings suggest that ejectives and implosives may have different phonological representations (gestural scores) that cannot be explained by simple bi-directional distinctions of egressive and ingressive air flow or by the mere presence of larynx raising/lowering. For example, ejectives are characterized by larger upward movement of the larynx *as well as* tighter coordination of oral-larynx timing compared to pulmonic stops and implosives. And implosives are distinguishable from pulmonic counterparts in terms of the timing relations between oral and vertical larynx gestures but not in the spatiotemporal actions of their individual gestures.

This specific contrast in *intergestural timing* between voiced stops and voiced implosives highlights the significance of multi-gesture complexes’ inherent coupling structures. Segments, at least those with multiple coupled gestures, are represented not only with regard to the types of compositional gestures and the degree of gestural activations (or magnitude) but also with regard to the temporal arrangement or phasing among the gestures. The current study proposes that linguistic contrasts can be manifested in the temporal organization between coordinated gestures as well as in the *stability* in intergestural timing, for multi-gestural molecules.

The findings suggest that the segment-internal intergestural lags for ejectives are more stable than for implosives. Such a coordination distinction can be modeled by phase windows or some similar approach allowing variability in intergestural phasing relations, representing ejectives with a tighter relationship between larynx raising and oral gestures than implosives’ larynx lowering and oral gestures (Byrd 1996a; Saltzman and Byrd 1999, 2000). Specifically we propose, referring back to the coupling schema in Figure 1, that the presence of multiple coupling relations between oral gestures and larynx *raising* gestures and the single coupling between oral and larynx *lowering* gestures predict that intergestural timing is more stable in ejectives compared to implosives (see also Nam et al. 2009 for discussion of more coupling links leading to less variability). Byrd (1996b) discusses another non-pulmonic consonant – click – saying that “in certain cases a precise temporal coordination may be necessary to yield aerodynamic properties that typify a sound, such as ingressive airflow in a click” (p. 160). She predicts that the coordination of larynx raising/lowering gestures with oral gestures in ejectives/implosives should be more stable than the coordination of those gestures with adjacent vowels.

Oral-vertical larynx timing is also shown to be less variable in non-pulmonic consonants than in pulmonic ones. We attribute this to their specific coupling graphs having no direct C-C coupling between oral and laryngeal gestures for pulmonic consonants, with only weak indirect C-V coupling relations present. Thus, it may be both the number of direct links *and* the coupling strengths that differentiate the timing stability between non-pulmonic and pulmonic consonants.

In addition to an examination of temporal lags among the constituent gestures of these consonant segments, the findings for the relative timing patterns in light of durational changes in the individual components crucially shows that the intergestural timing in question is not affected by gestural duration in implosives compared to the systematic variation of intergestural timing over changes in gestural duration found for the voiced stops. Similar results are found in Shaw et al. (2019) showing that the timing in complex segments is not affected by gestural duration, but that the timing in consonant sequences varies with changes in gestural duration. The present investigation illuminates how the covariance relations between timing and duration can be used to understand the rigidity or flexibility in timing for multi-gestural phonological units. When the timing of the two (or more) articulatory gestures is critical, other gestural actions (e.g., gestural duration) may compensate to preserve the relative timing. Such stable relative timing may indicate that a specific temporal coordination must necessarily be represented phonologically, for example, in the coupling structure. Implosives’ stability in timing across durational variations suggests that voiced implosives and voiced plosives are differentiated not only by timing patterns but also by the cohesion of intergestural timing, which can be represented by the topology of the coupling graphs and/or coupling strength.

In sum, the current investigation of Hausa ejectives, implosives, and pulmonic consonants highlights the important role of the vertical larynx gesture in creating phonological contrasts both in terms of larynx raising magnitude (ejectives vs. voiceless pulmonic consonants) and in terms of intergestural timing (implosives vs. voiced pulmonic counterparts). Based on the spatiotemporal characteristics of multi-gesture complexes, timing (phasing relations) of the gestures, in addition to duration (activation interval) and magnitude (target), must be encoded in the representation of these complex segmental gestural molecules. Additionally, we propose that the stability of the timing relations within the multi-gesture complex must also be captured by the phonological representation. A coupling graph provides a mechanism for doing so, enabling the prediction of differential stability in relative timing based on the coupling architecture.

One of the limitations in the current experiment is that the comparisons between ejectives and implosives are not based on the minimal pairs because Hausa ejectives and implosives are not produced with identical places of articulation. To ensure direct comparisons between ejectives and implosives, future articulatory studies on non-pulmonic consonants should investigate languages such as Zulu (includes bilabial and velar ejectives and implosives) and Yucatec Maya (includes bilabial ejectives and implosives). Investigation of the stricture (or closure) in the larynx, together with vertical larynx actions, will provide timing of larynx raising/lowering with accompanying constriction of the larynx. Such articulatory information stands to strengthen the connection between the dynamics of larynx actions and their aerodynamic consequences affecting pressure differentials. Furthermore, adding another level of prosodic conditions (i.e., utterance-initial [post-pausal] or at an Accentual Phrase boundary) to supplement the Intonational Phrase condition included here could reveal potentially gradient directional behaviors (e.g., in vertical larynx magnitude and/or gestural overlap) for different types of non-pulmonic consonants as well as pulmonic ones. Complex gestural molecules can be further examined in the context of a languages’ system of contrasts to assess whether, for example, the stability of larynx-oral coordination patterns in line with velum-oral coordination; manipulation of syllable structure or perturbation studies of coordination within multi-gesture complexes could be deployed to this end. Finally, the articulatory findings on the non-pulmonic consonants can be further supported by detailed description of aerodynamic mechanisms along with concurrent aerodynamic and acoustic measurements, which are left for future studies.

## 5 Conclusions

The current work presents an investigation of the spatiotemporal properties of vertical larynx movement in Hausa consonants and of how those actions are coordinated with supralaryngeal constriction gestures. The results of this rtMRI articulatory study suggest that phonological contrasts between non-pulmonic consonants and pulmonic consonants are manifested with different gestural sequencing patterns as well as with different timing variability. In addition to ejectives and implosives exhibiting raising and lowering of the larynx, respectively, the larynx gestures’ vertical position differs for different consonant types (ejective fricatives > ejective stops among ejectives and ejectives > implosives, voiced obstruents). Moreover, the beginning of the vertical larynx movement is tightly locked to the closing achievement of the oral gesture in non-pulmonic consonants, with ejectives exhibiting a more stable larynx-oral coordination than implosives.

The results also highlight that contrast among these segmental multi-gesture complexes includes not only a specification of the vertical laryngeal gesture and the glottal adduction gesture but also a specification of intergestural timing and stability relative to the oral constriction gestures. For instance, voiced implosives and voiced plosives contrast in intergestural timing and its variability, rather than in terms of the spatiotemporal properties of individual gestural actions. Therefore, the representational encoding of coupling relations and the consequent variability of the coupling graphs may be crucial to differentiating non-pulmonic consonants from pulmonic counterparts. How much of this additional specification needs to be stipulated in a phonological representation, and how much can follow automatically from a segment-internal dynamics is an open question for the future. Overall, the current study deepens our understanding of the role of vertical larynx actions and their coordination with oral gestures in consonants and helps advance modeling of the articulatory representation of complex multi-gestural molecules.
